# Bromide of Potassium in Leucorrhœa

**Published:** 1870-11

**Authors:** A. H. Kinnear

**Affiliations:** Metamora, Ill.


ARTICLE V. — *Bromide of Potassium in Leucorrhœa.* By A. H. KINNEAR, M.D., Metamora, Ill.

What I have to say, upon this subject, may not be altogether new to the profession at large, but as I have seen nothing of the kind in the medical periodicals, I have deemed my experience sufficiently interesting to warrant me in laying it before the readers of the JOURNAL.

Some two years ago, I was treating a case of leucorrhœa of some two years' standing. The remedies that I was prescribing in the case, were those that are usually recommended in works on Gynæcology, but after a fair trial of them, and failing to meet with the success I desired, I determined to change my treatment. A little previous to this time, I had occasion to prescribe Bromide of Potassium to a gentleman who was, and had been for a long time, troubled with gleet; but the prescription was given to procure sleep and allay nervousness. The prescription was continued for one week. At the expiration of this time he reported to me, and asked if I intended that medicine to cure the gleet, which he said it had accomplished. From this circumstance I was led to believe that it might be a valuable remedy in leucorrhœa, and, in fact, in all diseases of the genitalia, more particularly such affections as result from congestion of the mucous membrane of the genital tract, and as I had a case on hand which would test its virtues, I immediately prescribed it to my lady patient—twenty grs. in solution, twice each day, thirty minutes after meals. In ten days' time my patient reported herself as feeling much better; the discharge was lessened, she could sleep better, appetite was improving, and, in fact, there was decided improvement in every particular. I continued the prescription four weeks longer, omitting it occasionally for a short interval on account of the stomachic irritation it produced. At the expiration of this time all symptoms of her complaint had disappeared, and up to the present time she has had no return of the affection.

Since that time I have kept notes of twelve marked cases of leucorrhœa, none of which were of less than six months, and some of two years, standing when they applied to me for treatment. The prescription in each case was Bromide of Potassium in solution, the dose according to the severity of the case, and two months being as long as the treatment was required in any case. The majority of the cases yielded to the treatment in four weeks.

I found that it mattered not whether it was vaginal or uterine leucorrhœa; both seemed to yield with equal readiness to the remedy. I am satisfied, from the short experience I have had, that Bromide of Potassium will give better satisfaction in arresting this trouble, and all kindred complaints, than any remedy we have. We obtain, as it were, two effects from the use of this drug: the alterative and nervo-sedative; producing a marked sedative effect upon the genital organs; allaying all irritation of the parts, in fact, putting them at rest; thus giving the medicine every chance to produce its alterative effect on the parts diseased.

The question may arise in the minds of some of your readers, was it not necessary to give tonics in conjunction with this medicine? I found that in the majority of the cases the Bromide of itself soon created sufficient appetite for all the nourishment that was necessary in the case. In administering this remedy in leucorrhœa, the dose must be large enough to have a marked impression on the system, or else it will be liable to fail in accomplishing the desired effect.

